# Neck Pain Disability on Headache Impact and the Association between Sleep Disturbance and Neck Pain in Migraine

**DOI:** 10.3390/jcm12123989

**Published:** 2023-06-12

**Authors:** Hee-Jin Im, Yoo-Ha Hong, Soo-Jin Cho

**Affiliations:** Department of Neurology, Dongtan Sacred Heart Hospital, Hallym University Medical Center, Hwaseong 18450, Republic of Korea; coolere@naver.com (H.-J.I.); dbgk486@naver.com (Y.-H.H.)

**Keywords:** neck pain, migraine, primary headache, headache frequency, headache burden

## Abstract

Neck pain (NP) is a prevalent symptom among migraine patients, but its disability on headache impact and the contributing factors for comorbid NP are poorly understood. This study aimed to investigate NP disability on the impact of headaches among migraineurs and factors linked to comorbid NP, including sleep-related variables. This cross-sectional study was conducted at a university hospital headache center, for headache patients at their first visits. Included in the study were 295 patients with migraines (217 females; 39.0  ±  10.8 years; 101 chronic migraine). Information on NP, history of physician-diagnosed cervical spine or disc disorders, detailed parameters of headache, and sleep and mood variables were collected. Logistic analysis of the severe impact of headache and contributing factors for NP were performed. NP was present in 153 participants (51.9%) with migraine, with high NP disability observed in 28 patients, and 125 patients had low NP disability. In multivariable analysis, NP disability, medication days per month, severe disability of migraine, and excessive daytime sleepiness were significant predictors for severe impact of headache. Thirty-seven patients with physician-diagnosed cervical spine or disc disorders were excluded from the NP analysis. Higher monthly headache days, female gender, and a high likelihood of obstructive sleep apnea were positively correlated with the presence of NP among migraineurs in multivariable analysis. Overall, the study highlights the potential impact of sleep-related variables and monthly headache days on NP in these patients. The high disability of NP was also associated with severe impact of headache.

## 1. Introduction

Headache disorder and neck pain (NP) were the fifth and 19th causes of disability-adjusted life years, respectively, among the highly active ages of 25–49 years, respectively, in the Global Burden of Disease Study 2019 [1,2]. Migraine accounts for the majority of disabling headache disorders, so the association of migraine and neck pain can cause a substantial burden in the highly active age group [3]. Migraine can lead to NP even without cervical pathology due to the convergence of the trigeminal branch and cervical spinal segments, and vice versa [4]. Although study designs were heterogeneous in terms of exclusion criteria for cervical spine pathology or secondary headache, such as medication-overuse headache, NP was reported in 77% of migraineurs, 87% of chronic migraine patients, and 23% of controls without headache in a meta-analysis [5,6,7]. However, the parameters of NP, such as disability, severity or duration, and associating factors of comorbid NP has not been well evaluated in patients with migraine. Sleep disturbances and sleep disorders are well-known triggering factors among migraine patients, but their effects on NP in migraineurs have not yet been evaluated either [8]. Recent evidence has demonstrated that insomnia and sleep deprivation were associated with NP development in approximately half of the patients (53.6% and 42.2%) with chronic NP; it has also been linked to a high pain intensity [9,10].

In addition, the exclusion of cervicogenic headache in the NP study in migraine is problematic. It is difficult to define the onset of the disease in this prevalent cervical disorder. The prevalence of cervicogenic headache was reported as 0.17% in the general population and frequently cooccurred with migraine or medication overuse. In previous studies, 6–20% of patients with cervical stenosis or disc herniation and spinal surgery presented with headache, and the atypical presentation suggested a worse outcome of post-surgical headache [11,12]. Therefore, a significant proportion of headaches in patients with cervical pathology may be associated with migraine or primary headache disorders rather than cervicogenic headache. This study aimed to evaluate the impact of headaches in relation to the disability of NP and to investigate associating factors for the presence of NP without the physician-diagnosed cervical disorder among migraineurs in a headache clinic of a university hospital.

## 2. Materials and Methods

### 2.1. Participants

Participants who met the inclusion criteria were recruited from August 2020 to December 2021 at the Headache Clinic in the Department of Neurology of Hallym University Dongtan Sacred Hospital (Hwaseong, Republic of Korea). This study was a cross-sectional study carried out at a single university hospital. The inclusion criteria for participants were as follows: (1) participants were assessed at their very first visit, (2) over 19 years of age, and (3) diagnosed with migraine. The exclusion criteria were as follows: (1) participants that had a headache type other than migraine in main nature, (2) significant neck trauma or post-traumatic headache, (3) participants with missing information on NP, (4) participants that were cognitively not able to participate in the interview, and (5) participants that had a language or intellectual dysfunction. All participants were interviewed and evaluated by two neurologists (HJ Im and SJ Cho) using a self-administered questionnaire and clinical and neurological examinations were performed. Informed consent was obtained from all patients to be included in the study and this study was approved by the institutional review board of Hallym University Dongtan Sacred Hospital (no. 2020-04-008). The patient data used in this study contained no personal or identifying information. All individuals agreed to participate in a headache interview and a clinical examination. All methods were performed in accordance with the relevant guidelines and regulations of the Declaration of Helsinki.

### 2.2. Assessment of Headache

The diagnosis of migraine was determined by two neurologists through the individual interview based on the diagnostic criteria of the third edition of the International Classification of Headache Disorders (ICHD-3) [13]. The following were also evaluated: headache days per month, medication days per month, Headache Impact Test (HIT)-6 for the impact of a headache [14], and Migraine Disability Assessment (MIDAS) scores for disability of migraine [15]. Severe impact of headache was defined as a HIT-6 score ≥ 60. Severe disability of migraine was defined as a MIDAS score ≥ 21. Medication-overuse headache (MOH) was defined by the diagnostic criteria of ICHD-3.

### 2.3. Assessment of NP

The presence of NP was assessed by a self-administered questionnaire. Participants were asked if they had NP and physician-diagnosed cervical spine or disc disorder. The choices were Yes or No. If the response was yes for NP, several questions about NP features were asked. The participant self-graded the severity of NP as low disability/low intensity (grade 1), low disability/high intensity (grade 2), high disability-moderate limiting (grade 3), and high disability/severely limiting (grade 4) according to the recommendation for survey by the Bone and Joint Decade 2000–2010 Task Force on Neck Pain and its Associated Disorders [16,17]. The duration of NP was classified as transitory (less than 1 week), short duration (1 week or longer, but less than 3 months), and long duration (3 months or longer). Participants were asked to prioritize pain and the duration of NP. They answered, “In general, which one would you rate more severe for your pain, headache or NP or both?” to distinguish NP from NP associated with the headache attack itself and NP outside the headache attack the participants were also asked, “Is your NP associated with the headache attack? If yes, what proportion among headache attacks?”

### 2.4. Assessment of Sleep and Mood Parameters

Excessive daytime sleepiness was defined as an Epworth Sleepiness Scale score ≥ 11 [18]. The likelihood of obstructive sleep apnea was assessed by the STOP-bang questionnaire. A score over 3 reflected a moderate risk of sleep apnea during sleep [19]. Insomnia symptom was evaluated using the Insomnia Severity Index with a score >14 reflecting moderate-to-severe insomnia symptoms [20]. To assess the presence and severity of a depressed mood, the Patient Health Questionnaire-9 was used. A total score of ≥10 indicated the presence of a significant depressed mood [21]. Anxiety was indicated by a Generalized Anxiety Disorder-7 and a total score of ≥6 indicated the presence of anxiety.

### 2.5. Statistical Analysis

Categorical data were presented as frequencies and percentages (%). Continuous data, such as age, monthly headache days, and MIDAS score were presented as the mean and standard deviation or median (quartile) according to the normality of variables by the Shapiro test. For group comparisons between those with severe disabling NP, mild disabling NP, and those without NP, either the Kruskal–Wallis test, analysis of variance, the chi-square test, or Fisher’s exact test was used for continuous or categorized variables depending on the normality of variables and frequencies of each cell, respectively.

Univariable and multivariable logistic regression analyses were used to test the NP disability and other predictive factors for the severe impact of headache (HIT-6 ≥ 60). Data analysis was performed using R for Windows (ver. 4.1.2; R Foundation for Statistical Computing, Vienna, Austria) and R Studio (ver.2021.02.0 + 443; R Studio, Boston, MA, USA). To test the association between NP and contributing factors, multivariable logistic regression analysis was used with headache and sleep-related parameters as well as age, sex, BMI, depression and anxiety severity score after excluding patients with physician-diagnosed cervical spine or disc disorder. Regression analysis was performed using SPSS version 24 (SPSS, Chicago, IL, USA), and *p* < 0.05 indicated statistical significance.

## 3. Results

### 3.1. Participants’ Characteristics

**Figure 1:**
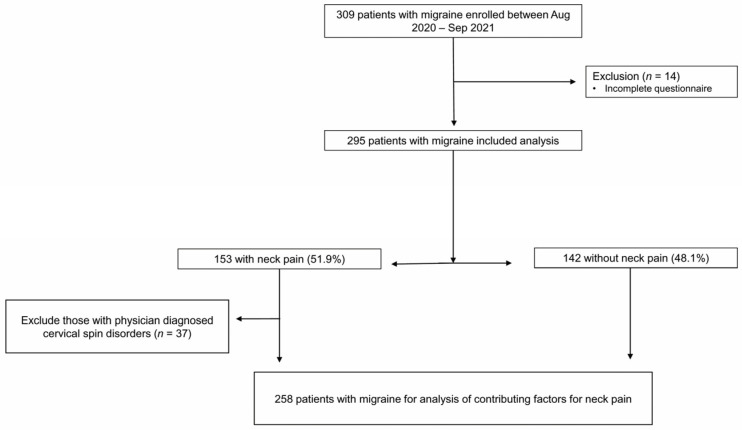
Flow chart of patient selection for analysis.

A total of 309 participants with migraine were enrolled in the study. After excluding 14 participants with missing information about NP, the remaining 295 participants were included in our analysis. The participant flowchart is represented in Figure 1. The average age of the participants in the study was 39.0 ± 10.8 years (range 19–81 years). There were 217 female participants (73.6%). There were 101 (34.2%) participants with chronic migraine and 37 (12.5%) participants with migraine with aura. Monthly headache days were 11.5 ± 8.6 days and the HIT-6 score was 59.8 ± 8.1.

NP was present in 153 (51.9%) participants with migraine. Physician-diagnosed cervical spine or disc disorders were reported in 37 (12.5%) patients (Figure 1).

NP disability was categorized as low in 125 (grade 1, 83 and grade 2, 42) and high in 28 patients (grade 3, 17 and grade 4, 11). Duration of NP was less than 1 week in 74 patients, between 1 week to 3 months in 36 patients, and more than 3 months in 43 patients. As for severity, 104 patients (68.0%) answered that their migraine was more severe than their NP, 33 patients (21.5%) answered that both were severe, and 16 patients (10.5%) answered that their NP was more severe than their migraine. NP was associated with a headache in 117 patients (76.5%) and was not associated with a headache in 36 patients (23.5%). Patients with NP-associated headache frequently reported more severe NP disability than the patients with NP was not associated headache (26/117, 22.2% vs. 2/36, 5.6%, *p* = 0.026). 

Patients with NP were more often female, and had more monthly headache days and a higher proportion of MOH, migraine disability, and severe impact of headache than those without NP (Table 1).

### 3.2. Disability of NP as the Predictor for Severe Impact of Headache in Migraineurs

To evaluate the influence of NP disability, univariable and multivariable logistic analyses were performed. NP disability, chronic migraine, medication-overuse headache, monthly headache days, medication days per month, severe disability of migraine (MIDAS ≥ 21), anxiety (GAD-7 ≥ 6), depressive mood (PHQ-9 ≥ 10), excessive daytime sleepiness (ESS ≥ 11), and moderate to severe insomnia (ISI ≥ 15) were significant predictors for the severe impact of headache (Table 2). In multivariable analysis, NP disability, medication days per month, severe disability of migraine (MIDAS ≥ 21), and excessive daytime sleepiness (ESS ≥ 11) were significant predictors for the severe impact of headache. Physician-diagnosed cervical spine or disc disorders were not significant predictors for the severe impact of a headache in multivariate logistic analysis (Table 2).

### 3.3. Contributing Factors for the Presence of NP in 258 Migraine Patients after Excluding Those with Physician-Diagnosed Cervical Spine or Disc Disorders

Considering different pathophysiology depending on whether cervical or disc disorders could lead to confusing analyses, the association between the presence of NP and contributing factors in migraineurs was accessed by a multivariate regression analysis in 258 participants after excluding physician-diagnosed cervical spine or disc disorders (Figure 1 and Table 3). Females are significantly associated with the presence of NP with an Odds ratio of 1.92 (CI 1.02–3.61, *p* = 0.042) and monthly headache frequency was also correlated with borderline significance (OR 1.04, CI 1.00–1.08, *p* = 0.059). Sleep-related factors demonstrated a higher OR than headache-related factors for NP. Having a high risk of obstructive sleep apnea remained significantly associated with the presence of NP (OR 3.61 and 3.31, 95% CI 1.19–11.00, *p* = 0.024). Other factors like chronic migraine, medication frequency and impact of headache did not show significant associations of the presence of NP.

## 4. Discussion

This study yielded several key findings: (1) approximately half (51.9%) of the patients with migraine experienced NP; (2) NP were associated with headache attacks in 76.5% of migraineurs with NP and they reported severe NP disability compared to the patients with NP not associated headache (22.2% vs. 5.6%, *p* = 0.026); (3) significant predictors for the severe impact of headache were NP disability, medication days per month, severe disability of migraine, and excessive daytime sleepiness among total participants; (4) 12.5% reported physician-diagnosed cervical spine or disc disorders, but were not significant predictors for the severe impact of a headache in multivariate logistic analysis; (5) in multivariable analysis after excluding those with physician-diagnosed cervical spine or disc disorders, NP was more prevalent in relation to females, headache frequency (monthly headache days), and a high likelihood of obstructive sleep apnea.

The prevalence of NP in patients with migraine was reported at 58.6–81.5% in different study populations (general population, headache clinic) mostly based in Western countries [3,7,22,23]. However, a relatively lower frequency of NP in our study (about 50%) may be partly attributed to the racial difference or study design. According to the global age-standardized rates in 2019, the prevalence of NP was 2697 globally and 1719 in the Republic of Korea per 100,000 population, respectively [3].

NP was frequently reported as a trigger, prodrome, or accompanying symptom of migraine [7]. The prevalence of NP was reported by 42.8% for mild migraine pain, 61.1% for moderate migraine pain and 72.6% for severe migraine pain. NP is also more frequently associated with migraine than nausea, and its high association rate with headache and disability suggests that it is an accompanying symptom of migraine [6,24].

Our study showed that the NP disability was an important predictor for severe impact of headache, hence, detailed information about NP is more important than the presence of NP itself. The association with a headache on the severity of NP in this study is consistent with the result from a study regarding Neck Disability Index. The study reported Neck Disability Index reflects allodynia and HIT-6 but not cervical musculoskeletal dysfunction in migraine [25].

The convergence of cervical and trigeminal nociceptive afferents at the trigeminocervical complex can result in an NP from trigeminal activation by migraine or vice versa [26]. Furthermore, increased expression of calcitonin gene-related peptide (CGRP) gene in temporal relation to inflammation of cranial muscles in experimental models [27] suggests that CGRP also plays a role in muscle nociception in migraines and it is related to a higher NP prevalence as well as more severe migraine symptoms.

The association between sleep disorders and NP in this study is likely to be a general trend of chronic pain disorders rather than a specific association in migraine. Obstructive sleep apnea (OSA) is also associated with the development and progression of many painful conditions, such as headaches, temporomandibular disorders, and fibromyalgia [28]. OSA was reported to be a risk factor for increasing subjective pain intensity and lowering pain tolerance and thresholds [29], which may be attributed to potent pro-inflammatory mediators, such as C-reactive protein, TNF-α, IL-1β, IL-6, and CGRP which was also involving pain modulation, especially in migraine [30]. Sleep disorders such as OSA result in hypoxia and sleep fragmentation, following changes in sleep architecture (e.g., increased light sleep, decreased slow-wave sleep, decreased REM sleep and changes in total sleep time) [31]. Sleep fragmentation, intermittent hypoxia and its consequent inflammation modulate pain perception and exacerbate hypersensitivity for musculoskeletal pain, as proven in an animal model [32], as well as daytime sleepiness or impaired daytime performance.

Regarding sleep and migraine shares anatomical structures and neuropeptide involved in both regulation [33], individuals who suffer from migraines tend to be more sensitive to the headaches caused by obstructive sleep apnea (OSA), which can result in a greater overall headache burden in relation to a lower tolerance for the hypoxemia-induced headaches associated with OSA [34]. However, the rates of OSA are not conclusively higher in individuals with migraines. Although the direct link between OSA and NP or migraine is uncertain, the association between sleep disorders and NP among migraineurs might be reasonable.

## 5. Strengths and Limitations

To the best of our knowledge, this is the first study to evaluate the prevalence and disability of NP in a clinical migraine population in Asia. The proportion of migraine with physician-diagnosed cervical spine or disc disorders was not small in a headache clinic, but the influence of the severity of headache was not evident in this study. An additional interview with a comprehensive questionnaire was conducted to assess the NP characteristics, including sleep related, mood and migraine factors. This study found a clear association between NP and sleep disturbance, such as insomnia and sleep apnea, as well as migraine frequency.

However, this study has some limitations. First, participants of the current study were recruited from a single headache center, so sampling bias cannot be avoidable. Second, a detailed evaluation for cervicogenic headache was not performed based on the diagnostic criteria [35,36], considering its various anatomical structures for neck pain. Differentiation of concomitant cervicogenic headache among migraineurs may be helpful for identifying the candidate for the invasive procedure or physical therapy [26] and further detailed exploration of the complexities of neck pain would be needed. Third, the pathologic entities of physician-diagnosed cervical spine or disc disorders, comorbid tension-type headache, or fibromyalgia were not defined. Forth, sleep parameters for sleep disturbance were self-administrated and sleep apnea, especially, was not obtained from objective measurements through polysomnography. Nonetheless, our findings provide valuable insight into the relationship between NP, sleep disturbance, and migraine burden, this evidence is based on participants’ self-reported profiles in regards to their sleep habits and headache severity. Further extensive and objective longitudinal research is required in the field.

## 6. Conclusions

NP was present in half of the participants with migraine and NP with a headache was related to the severe disability of NP. Migraineurs with high NP disability were associated with severe impact and disability of migraine along with depressive mood, and daytime sleepiness. After adjustment of headache parameters and emotional variables, NP disability was the significant predictor for the severe impact of migraine. Additionally, comorbid sleep disorders such as obstructive sleep apnea, can be associated with NP in the context of migraines and may contribute to the progressive worsening of headaches.

## Figures and Tables

**Table 1:** Comparison of clinical features according to neck pain among patients with migraine (*n* = 295).

Characteristics	Presence of Neck Pain (*n* = 153)	Absence of Neck Pain (*n* = 142)	*p*-Value
Age (y)	39.9 (32, 47)	38.0 (31, 44)	0.166
Female sex	123 (80.4)	94 (66.2)	0.009
BMI (kg/m^2^)	22.9 (20.8, 24.9)	23.2 (20.9, 25.0)	0.373
Chronic migraine	61 (39.9)	40 (28.2)	0.046
Migraine with aura	15 (9.8)	22 (15.5)	0.194
Monthly headache days	12.8 (5, 20)	10.1 (4, 15)	0.006
Medication day per month	7.6 (2, 10)	6.4 (2, 10)	0.261
Medication overuse headache	48 (31.4)	28 (19.7)	0.031
MIDAS ≥ 21	70 (45.8)	42 (29.6)	0.006
Severe impact of headache (HIT-6 ≥ 60)	101 (66.0)	71 (50.0)	0.008
Anxiety (GAD-7 ≥ 6)	67 (43.8)	56 (39.4)	0.522
Depressive mood (PHQ-9 ≥ 10)	48 (31.4)	30 (21.1)	0.063
Excessive daytime sleepiness (ESS ≥ 11)	32 (20.9)	26 (18.3)	0.677
Moderate-to-severe insomnia (ISI ≥ 15)	40 (26.1)	23 (16.2)	0.052
High risk of obstructive sleep apnea (STOP-bang ≥ 3)	16 (10.5)	6 (4.2)	0.070

The data are presented as median (quartile) according to normality of variable. Categorical variables are presented as number (%) and these *p*-values are based on the chi-square or fisher exact test. BMI, body mass index; HIT-6, Headache Impact Test-6; MIDAS, Migraine Disability Assessment; GAD-7, Generalized Anxiety Disorder-7; PHQ-9, Patient Health Questionnaire-9; ESS, Epworth Sleepiness Scale; ISI, Insomnia Severity Index; STOP-bang, STOP-bang Questionnaire.

**Table 2:** Disability of neck pain and other predictive factors for severe impact of headache (HIT-6 ≥ 60) in migraineurs (*n* = 294) *.

	Univariable		Multivariable	
	OR (95% CI)	*p*-Value	OR (95% CI)	*p*-Value
Disability of neck pain				
Absence of neck pain	1		1	
Low disability of neck pain	1.55 (0.95–2.52)	0.077	1.10 (0.56–2.16)	0.785
High disability of neck pain	8.33 (2.41–28.85)	0.008	6.14 (1.41–26.70)	0.016
Age	0.98 (0.96–1.00)	0.108	0.98 (0.95–1.01)	0.213
Female sex	1.19 (0.71–2.01)	0.507	1.03 (0.50–2.10)	0.946
BMI	0.98 (0.92–1.06)	0.663	0.97 (0.88–1.07)	0.575
Chronic migraine	3.61 (2.09–6.21)	<0.001	0.97 (0.39–2.45)	0.953
Migraine with aura	0.82 (0.41–1.64)	0.575	1.38 (0.56–3.39)	0.484
MOH	5.48 (2.80–10.72)	<0.001	2.40 (0.83–6.91)	0.106
Monthly headache days	1.09 (1.05–1.13)	<0.001	0.99 (0.94–1.04)	0.765
Medication day per month	1.15 (1.09–1.21)	<0.001	1.10 (1.03–1.18)	0.008
MIDAS ≥ 21	11.48 (5.99–21.97)	<0.001	7.19 (3.42–15.14)	<0.001
Anxiety (GAD-7 ≥ 6)	2.19 (1.34–3.55)	0.002	1.07 (0.55–2.01)	0.844
Depressive mood (PHQ-9 ≥ 10)	4.16 (2.23–7.76)	<0.001	2.82 (1.14–7.00)	0.025
Excessive daytime sleepiness (ESS ≥ 11)	3.83 (1.89–7.74)	<0.001	2.57 (1.05–6.30)	0.038
Moderate to severe insomnia (ISI ≥ 15)	2.51 (1.35–4.69)	0.004	0.89 (0.35–2.24)	0.802
High risk of obstructive sleep apnea (STOP-bang ≥ 3)	2.00 (0.76–5.27)	0.161	1.53 (0.40–5.90)	0.536
Physician diagnosed cervical spine or disc disorders	1.81 (0.86–3.83)	0.118	1.45 (0.52–4.01)	0.479

* One case was deleted due to missing variables in medication days per month. OR, odds ratio; CI, confidence interval; BMI, body mass index; HIT-6, Headache Impact Test-6; MIDAS, Migraine Disability Assessment; GAD-7, Generalized Anxiety Disorder-7; PHQ-9, Patient Health Questionnaire-9; ESS, Epworth Sleepiness Scale; ISI, Insomnia Severity Index; STOP-bang, STOP-bang Questionnaire.

**Table 3:** Associative factors for presence of neck pain in migraineurs after excluding physician-diagnosed cervical spine or disc disorders (*n* = 258).

Factors		OR (95% CI)	*p*-Value
Demographic	Age	1.00 (0.97–1.03)	0.926
	Female sex	1.92 (1.02–3.61)	0.042
	BMI	0.96 (0.86–1.04)	0.294
Headache related	Chronic migraine	0.97 (0.51–1.86)	0.370
	Migraine with aura	0.71 (0.31–1.62)	0.415
	Monthly headache days	1.04 (1.00–1.08)	0.059
	Monthly medication days	0.93 (0.94–1.08)	0.370
	Severe impact of headache (HIT-6 ≥ 60)	1.55 (0.81–2.59)	0.215
Mood related	GAD-7 score	1.00 (0.92–1.08)	0.904
	PHQ-9 score	0.99 (0.92–1.08)	0.866
Sleep related	Moderate to severe insomnia (ISI ≥ 15)	1.86 (0.87–4.00)	0.112
	High risk of obstructive sleep apnea (STOP-bang ≥ 3)	3.61 (1.19–11.00)	0.024
	Excessive daytime sleepiness (ESS ≥ 11)	0.78 (0.38–1.63)	0.510

Data from multiple regression analysis were adjusted for age, sex, presence of weekend sleep extension and average sleep duration. OR, odds ratio; CI, confidence interval; BMI, body mass index; HIT-6, Headache Impact Test-6; GAD-7, Generalized Anxiety Disorder-7; PHQ-9, Patient Health Questionnaire-9; ESS, Epworth Sleepiness Scale; ISI, Insomnia Severity Index; STOP-bang, STOP-bang Questionnaire.

## Data Availability

If required, our data can be submitted.
